# Multiparametric MRI-Based Radiomics Model for Predicting H3 K27M Mutant Status in Diffuse Midline Glioma: A Comparative Study Across Different Sequences and Machine Learning Techniques

**DOI:** 10.3389/fonc.2022.796583

**Published:** 2022-03-03

**Authors:** Wei Guo, Dejun She, Zhen Xing, Xiang Lin, Feng Wang, Yang Song, Dairong Cao

**Affiliations:** ^1^ Department of Radiology, First Affiliated Hospital of Fujian Medical University, Fuzhou, China; ^2^ MR Scientific Marketing, Siemens Healthineers Ltd., Shanghai, China; ^3^ Department of Radiology, Fujian Key Laboratory of Precision Medicine for Cancer, The First Affiliated Hospital, Fujian Medical University, Fuzhou, China; ^4^ Key Laboratory of Radiation Biology of Fujian Higher Education Institutions, The First Affiliated Hospital, Fujian Medical University, Fuzhou, China

**Keywords:** diffuse midline glioma, H3 K27M mutant, multiparametric MRI, radiomics, machine learning

## Abstract

**Objectives:**

The performance of multiparametric MRI-based radiomics models for predicting H3 K27M mutant status in diffuse midline glioma (DMG) has not been thoroughly evaluated. The optimal combination of multiparametric MRI and machine learning techniques remains undetermined. We compared the performance of various radiomics models across different MRI sequences and different machine learning techniques.

**Methods:**

A total of 102 patients with pathologically confirmed DMG were retrospectively enrolled (27 with H3 K27M-mutant and 75 with H3 K27M wild-type). Radiomics features were extracted from eight sequences, and 18 feature sets were conducted by independent combination. There were three feature matrix normalization algorithms, two dimensionality-reduction methods, four feature selectors, and seven classifiers, consisting of 168 machine learning pipelines. Radiomics models were established across different feature sets and machine learning pipelines. The performance of models was evaluated using receiver operating characteristic curves with area under the curve (AUC) and compared with DeLong’s test.

**Results:**

The multiparametric MRI-based radiomics models could accurately predict the H3 K27M mutant status in DMG (highest AUC: 0.807–0.969, for different sequences or sequence combinations). However, the results varied significantly between different machine learning techniques. When suitable machine learning techniques were used, the conventional MRI-based radiomics models shared similar performance to advanced MRI-based models (highest AUC: 0.875–0.915 vs. 0.807–0.926; DeLong’s test, *p* > 0.05). Most models had a better performance when generated with a combination of MRI sequences. The optimal model in the present study used a combination of all sequences (AUC = 0.969).

**Conclusions:**

The multiparametric MRI-based radiomics models could be useful for predicting H3 K27M mutant status in DMG, but the performance varied across different sequences and machine learning techniques.

## Introduction

As a newly defined subtype of the 2016 WHO Classification of Tumors of the Central Nervous System, “diffuse midline glioma (DMG), H3 K27M mutant” is characterized by a genetic alteration pattern in either H3F3A or HIST1H3B/C (1). Compared to the wild-type group, the group with DMG with an H3 K27M mutation exhibited a particularly dismal prognosis, with 3-year overall survival of 5% and 2-year overall survival of less than 10% (2–5). In addition, the previous studies revealed that H3 K27M mutant status represented a potential novel therapeutic target for DMG, which confronts the fact of resistance to the conventional therapy strategies (6–10). Identifying H3 K27M mutant status plays an essential role in tumor diagnosis, survival prediction, and therapeutic decision-making. Surgical resection or biopsy could provide an accurate result of H3 K27M mutant status but is not always feasible due to tumor tissue’s spatial heterogeneity and unforeseeable complications. Developing a non-invasive method for accurately predicting H3 K27M mutant status is critical for DMG management.

Several recent attempts have been made to use the multiparametric MRI-based radiomics model to predict H3 K27M mutant status, but the results varied greatly (11–16). Most of them focused on different kinds of conventional MRI (cMRI), which could only reflect the tumor’s morphologic information and benefit limitedly to reveal tumor heterogeneity. The advanced MRI (aMRI) (e.g., diffusion-weighted imaging [DWI], susceptibility-weighted imaging [SWI], and dynamic susceptibility contrast perfusion-weighted imaging [DSC-PWI]), which could provide physiological information within the tumor, has been proved to be helpful in radiomics-based glioma genotype prediction (17–19). However, the utility of an advanced MRI-based radiomics model in predicting H3 K27M mutant status has not been well evaluated. On the other hand, previous studies indicated that the performance of the radiomics model predominantly varied with the type of image set used (20, 21). As such, it is unclear whether aMRI or a combination of cMRI and aMRI could make an equivalent or superior performance as compared to cMRI.

In addition to the heterogeneous sequence used, the previous studies on H3 K27M mutant status prediction employed a great diversity of machine learning techniques, including dimensionality-reduction algorithm, feature selector, and classifier. It has been well recognized that the radiomics model established *via* different machine learning techniques could achieve diverse results even when the same sequence was used (22, 23). This could be a potential reason for the inconsistent prior radiomics-based H3 K27M mutant status prediction results. Therefore, there is an urgent need for a head-to-head comparison of the prediction power across different machine learning techniques and sequence or sequence combinations to determine the best machine learning techniques with the best image sets.

The purposes of this study were to 1) detect the best MRI sequence or sequence combinations for predicting H3 K27M mutant status in DMG and 2) determine the optimal machine learning technique for different image sets.

## Materials and Methods

### Study Population

The Ethical Committee of the First Affiliated Hospital of Fujian Medical University approved this study. The requirement for written informed consent was waived due to the retrospective nature. One hundred two patients were consecutively enrolled in the present study from July 2010 to August 2021. The inclusion criteria were as follows: 1) patients have a pathological diagnosis of diffuse glioma and confirmation of H3 K27M mutant status; 2) tumor is located in the midline structure of the brain; and 3) full preoperative MR images were available. Exclusion criteria were as follows: 1) absence of any required MR images or the image quality was insufficient for analysis and 2) the tumor volume was less than 1.5 cm^3^. The patients were randomly split into training and test groups with a ratio of 7:3. Extra effort was made to keep the balance between the training and test cohorts.

### MRI Protocol

The neurologic MRI examinations were performed with the 3.0-Tesla MR scanner (MAGNETOM Verio/Skyra/Prisma, Siemens Healthcare, Erlangen, Germany). The standard multiparametric MRI sequences in the present study, including T2-weighted imaging (T2WI), T1-weighted imaging (T1WI), fluid-attenuated inversion recovery (FLAIR), contrast-enhanced T1WI (CE-T1WI), SWI, DWI, and DSC-PWI. The details of MRI acquisition parameters are listed in the **Supplementary Material 1** (**Table S1**). The apparent diffusion coefficient (ADC) map was automatically derived from DWI data with b-values of 0 and 1,000 s/mm^2^. The DSC-PWI raw data were scrolled into a dedicated commercial software package (SyngoVia, Siemens), and the standard perfusion maps (cerebral blood volume [CBV] and cerebral blood flow [CBF]) were conducted as guidance of the software. In the 4th phase during the DSC-PWI scanning, a standard dose (0.1 mmol/kg) of gadobenate dimeglumine (Gd-BOPTA) followed by 20 ml of saline was injected intravenously with a flow rate of 3 ml/s. CE-T1WI was scanned after DSC-PWI.

### Image Pre-Processing and Tumor Segmentation

Before pre-processing, the DICOM images were converted to the nifti format. The standard image pre-processing included four steps: 1) all sequences were registered to T2WI initially with a block matching algorithm; 2) following the co-registration, the images were resampled into the uniform voxel size of 1 × 1 × 5 mm^3^; 3) N4 Bias Field Correction package was applied to correct the bias filed; 4) finally, the image intensities were standardized to [0, 255] to reduce the influence of imaging intensity inconsistency. All of the pre-processing procedures were achieved using G.K software (Glioma kit, version 1.2.1.R, GE Healthcare, Shanghai, China).

Tumor segmentation was performed by one radiologist (DS, with 10 years of experience in neuroradiology) and verified by another radiologist (DC, with 30 years of experience in neuroradiology) who were unaware of the pathological results. The volume of interest (VOI) was created to cover the tumor core (including the enhancing, non-enhancing, and necrotic/cystic components) on T2WI with ITK-SNAP (http://www.itksnap.org) by referring to the T1WI, CE-T1WI, and FLAIR images. According to VASARI guidelines (Visually AcceSAble Rembrandt Images; https://wiki.nci.nih.gov/display/CIP/VASARI), the respective portions of the tumor were defined as described in the previous study (24, 25). As the radiomics feature extraction differed between VOIs, the intra-observer and inter-observer reproducibility analyses were achieved to minimize the influence of segmentation bias. Of intra-observer reproducibility analysis, the VOIs of 30 randomly chosen patients were segmented twice by one radiologist (DS). The inter-observer reproducibility analysis was performed based on the same cohort above, where the VOIs were segmented by two radiologists (ZX and DS, both with 10 years of experience in neuroradiology). The intraclass correlation coefficient (ICC) was calculated to evaluate the agreement of radiomics feature extraction.

### Radiomics Feature Extraction

An open-source software, FeAture Explore (V 0.4.2), was used for quantitative radiomics feature extraction with the Pyradiomics module on Python (3.7.6) (26, 27). A total of 851 features were extracted from each sequence image, consisting of 18 first-order statistics features, 14 shape-based features, 75 texture features, and 744 wavelet features from eight wavelet-transformed images (https://pyradiomics.readthedocs.io/en/latest/features.html). The details of the extracted features are listed in the **Supplementary Material 1** (**Table S2**). Eight sequences (T2WI, T1WI, FLAIR, CE-T1WI, ADC, SWI, CBV, and CBF) were used in the present study. Thus, a total of 6,808 features were extracted for analysis. We conducted 18 feature sets by the independent combination of features extracted from these eight sequences. The feature sets were generally named with the name of sequences. Especially, “cMRI,” “aMRI,” and “ALL” denote the combination of all cMRI sequences, aMRI sequences, and eight sequences, respectively.

### Radiomics Feature Matrix Pre-Processing

As described above, for the sake of minimizing the influence of VOI segmentation bias on radiomics feature calculation and further machine learning analysis, the features with an ICC value lower than 0.75 in either the intra-observer or inter-observer reproducibility analysis were removed. Then we applied the normalization to the remaining feature matrix. Three feature normalization methods were considered: mean normalization, min–max normalization, and Z-score normalization. The mean normalization subtracted each feature vector by the mean value of the vector and divided each feature by the length of the vector. For the min–max normalization, we rescaled the minimum and maximum values of the feature from zero to one. Then the feature vector was mapped to a unit vector. When the Z-score method was applied, we calculated each feature vector’s mean value and SD. Then each feature was subtracted by the mean value and was divided by the SD. Notably, only one normalization method was used in one machine learning pipeline.

### Radiomics Feature Dimensionality Reduction and Feature Selection

Since the feature space dimension was high, we applied two alternative feature dimensionality-reduction methods in the presented study, including Pearson’s correlation coefficient (PCC) and principal component analysis (PCA). The PCC was calculated for each pair of two normalized features, and we removed one of them if the PCC was larger than the preset threshold. By referring to the previous study, the threshold was set to 0.8 for the model using a single sequence and 0.6 for the model using a combination of different sequences (20). When the PCA method was chosen, the high dimension features were transformed into the relative lower dimension features. The feature vector of the transformed feature matrix was independent of each other.

Following feature dimensionality reduction, four optional methods were provided for feature selection, including ANOVA, recursive feature elimination (RFE), Kruskal–Wallis (KW), and Relief.

### Predictive Model Establishment

Seven machine learning classifiers were analyzed to determine the optimal model. The SVM classifier we used was based on a linear kernel function , and it may be more appreciated to be cataloged into the linear classifier. The sentence should be corrected as "These classifiers could be divided into three categories: linear (logistic regression [LR], linear discriminant analysis [LDA], and support vector machine [SVM]), non-linear classifiers (auto-encoder [AE] and decision tree [DT]), and ensemble classifiers (random forest [RF] and AdaBoost [AB]). The five-fold cross-validation was applied on the training dataset to determine the model’s hyper-parameter, such as the number of features and specific hyper-parameters of each classifier, which can be referred on the scikit-learn (https://scikit-learn.org/stable/index.html). The hyper-parameters were set according to the model performance on the cross-validation dataset.

**Figure 1:**
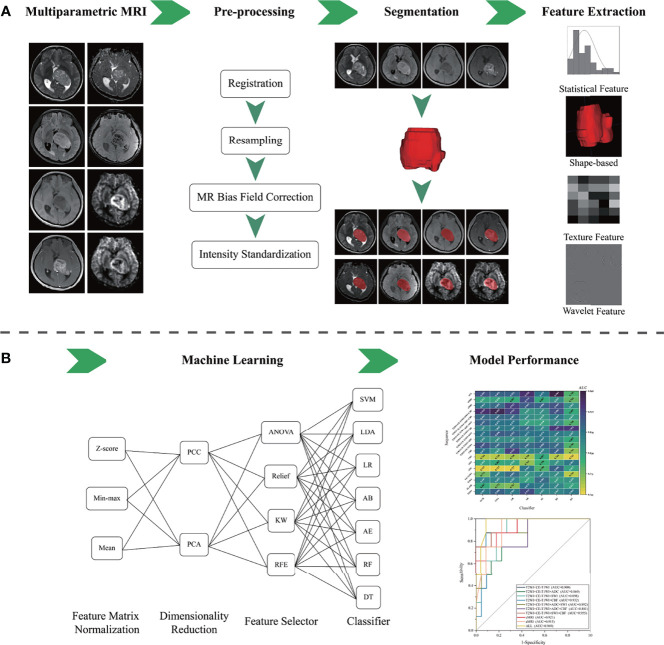
The flowchart of the presented study. **(A)** Multiparametric MRI data collection, image pre-processing, tumor segmentation, and radiomics feature extraction. **(B)** Machine learning and model performance analysis.

Considering different combinations of each procedure during model development, including sequence used, feature matrix normalization, dimensionality reduction, and feature selection, could provide controversial results with different classifiers. We analyzed models’ performance from 8 single sequences and 10 different sequence combinations with different machine learning techniques. Thus, a total of 3,024 models were conducted in the present study (18 [sequence groups] × 3 [feature matrix normalization] × 2 [dimensionality reduction] × 4 [features selector] × 7 [classifiers] = 3,024 [models]). The flowchart of the present study is illustrated in **Figure 1**. The above processes, including feature matrix normalization, dimensionality reduction, feature selection, and classifier fitness, were implemented with FeAture Explorer (V 0.4.2) on the training cohort. Then, we evaluated the models’ performance on the independent test cohort.

### Statistical Analysis

The performance of each model was evaluated with receiver operating characteristic curve analysis. The area under the receiver operating characteristic curve (AUC) and accuracy were calculated. We also estimated the 95% CI by bootstrap with 1,000 samples. To assess the variability in the performance of different models, we compared the top-one-performing models and the top-five-performing models of each sequence or sequence combination. Continuous variables of the baseline characteristics were described as the mean *±* SD and compared using the Mann–Whitney U test. Categorical variables of the baseline characteristics were described as number (percentage) and compared using Pearson’s chi-squared test. The comparison of AUCs between different models was performed using Delong’s test. The statistical analyses were performed with R statistical software (version 3.5.3; https://www.r-project.org/). A *p*-value <0.05 was considered statistically significant.

## Results

### Baseline Characteristics of Patients

Of the 102 patients, 27 (26.47%) patients were confirmed with an H3 K27M mutation. The mean age was 41.19 ± 20.64 years, and the male ratio was 64 (62.75%). No statistically significant difference was found in the baseline characteristics between the training and test groups (*p* > 0.05) (**Table 1**).

**Table 1:** Baseline characteristics of the training and test groups.

Characteristics	All (n = 102)	Training (n = 72)	Test (n = 30)	*p*-Value
Age (years)	41.19 ± 20.64	41.63 ± 20.80	40.13 ± 20.57	0.649
Gender (%)				0.711
Male	64 (62.75%)	46 (63.89%)	18 (60.00%)	
Female	38 (37.25%)	26 (36.11%)	12 (40.00%)	
H3 K27M mutant status (%)				0.977
Mutant	27 (26.47%)	19 (26.39%)	8 (26.67%)	
Wild type	75 (73.53%)	53 (73.61%)	22 (73.33%)	

A p-value <0.05 indicates the statistical significance of the variate difference between training and test sets. Continuous variables were described as the mean ± SD. Categorical variables were presented as the number, with percentages in parentheses.

### Performance of Sequence

**Figure 2:**
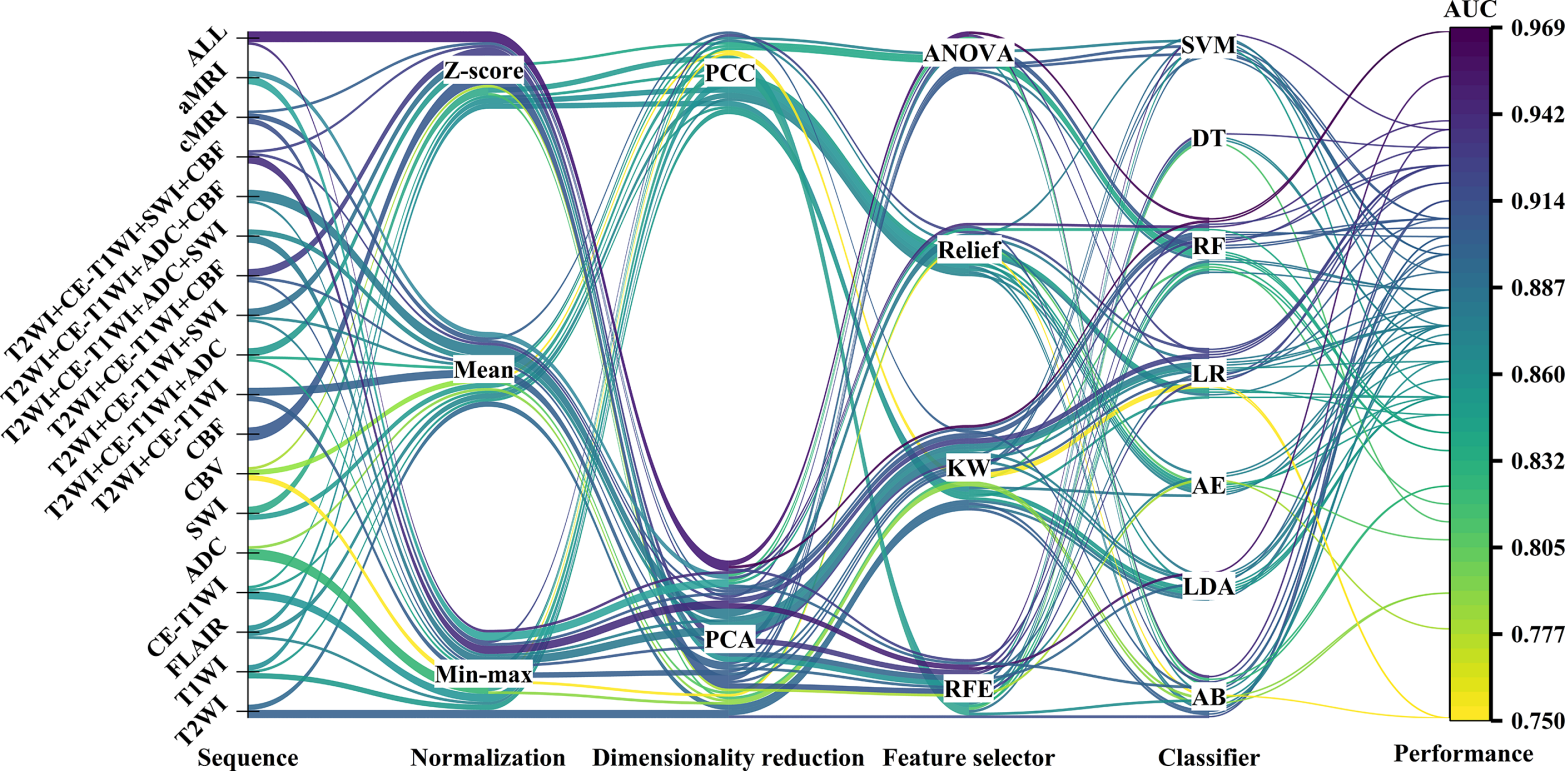
The machine learning pipelines and performance of top-five-performing models of different sequences. The color of lines indicated the performance of models in the test set.

**Figure 3:**
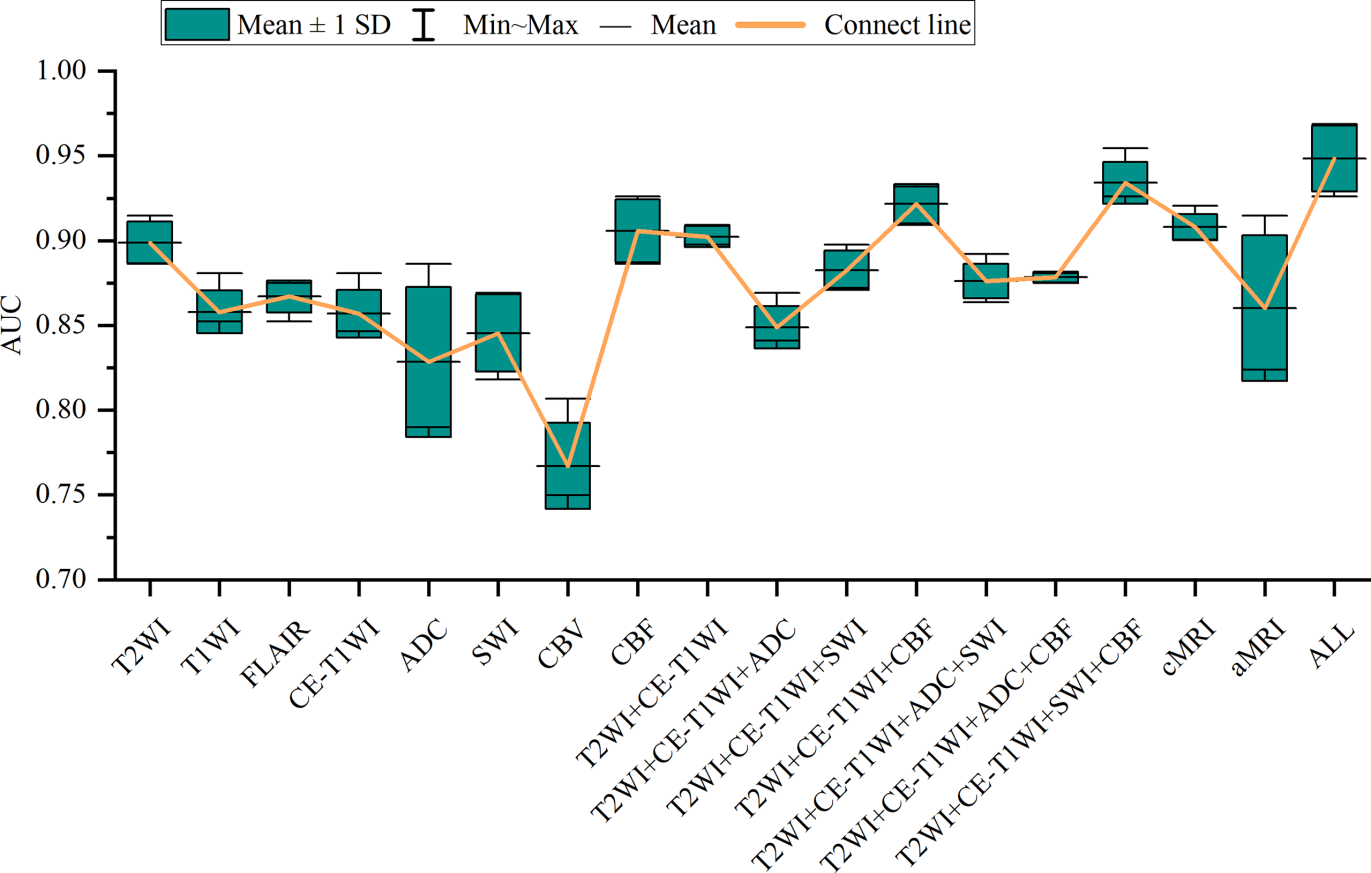
Box-and-whisker plots illustrate the top-five-performing area under the curve (AUC) values of different sequences.

**Figure 4:**
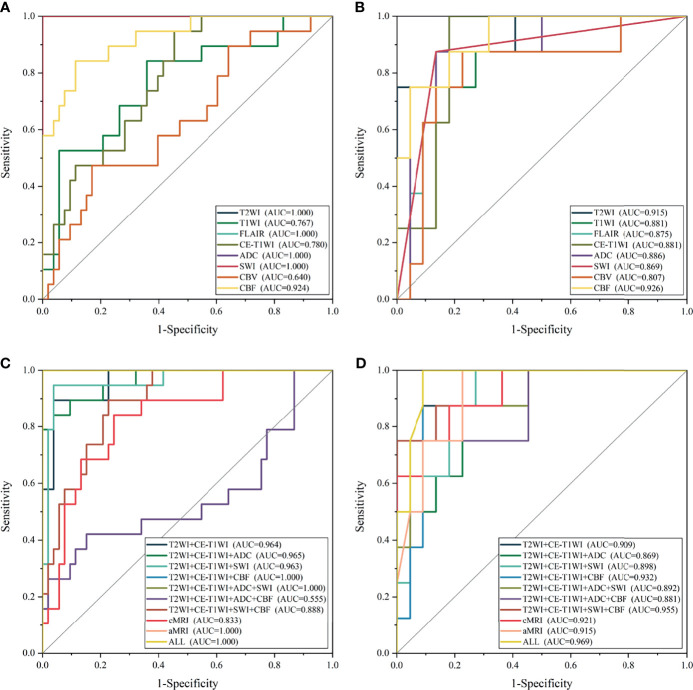
The receiver operating characteristic curve of the top-one-performing models of different sequences in the training **(A, C)** and test sets **(B, D)**.

In general, most of the high-performing models (with an AUC value larger than 0.9 in the test set) were conducted from the combination of different sequences (**Figures 2**, **3** and **Tables 2** and **S3**). The ALL model showed the strongest predictive power among various models for H3 K27M mutant status (AUC = 0.969), while the best single-sequence model was the CBF-based model (AUC = 0.926), followed by the T2WI-based model (AUC = 0.915). The CBV-based model yielded the lowest AUC value of 0.807 among the top-one-performing models of different sequences or sequence combinations (**Figure 4**).

**Table 2:** The performance of the top-one-performing models.

Sequence	Machine learning technique	Dataset	AUC	95% CI	ACC	SEN	SPE	PPV	NPV
T2WI	Min–max_PCA_RFE_AB	Training	1.000	1.000–1.000	1.000	1.000	1.000	1.000	1.000
		Test	0.915	0.769–1.000	0.900	0.750	0.955	0.857	0.913
T1WI	Z-score_PCC_KW_AE	Training	0.767	0.631–0.890	0.694	0.842	0.642	0.457	0.919
		Test	0.881	0.733–0.984	0.700	1.000	0.591	1.000	0.471
FLAIR	Mean_PCC_Relief_AB	Training	1.000	1.000–1.000	1.000	1.000	1.000	1.000	1.000
		Test	0.875	0.722–0.984	0.833	0.625	0.909	0.870	0.714
CE-T1WI	Min–max_PCC_Relief_LR	Training	0.780	0.669–0.883	0.653	0.947	0.547	0.429	0.967
		Test	0.881	0.733–0.984	0.800	1.000	0.727	1.000	0.571
ADC	Min–max_PCC_RFE_RF	Training	1.000	1.000–1.000	1.000	1.000	1.000	1.000	1.000
		Test	0.886	0.718–1.000	0.700	0.000	0.955	0.724	0.000
SWI	Mean_PCC_RFE_DT	Training	1.000	1.000–1.000	1.000	1.000	1.000	1.000	1.000
		Test	0.869	0.694–0.979	0.867	0.875	0.864	0.950	0.700
CBV	Mean_PCA_Relief_AE	Training	0.640	0.490–0.779	0.736	0.474	0.830	0.500	0.815
		Test	0.807	0.585–0.980	0.700	0.875	0.636	0.933	0.467
CBF	Z-score_PCA_RFE_LR	Training	0.924	0.844–0.983	0.875	0.842	0.887	0.727	0.940
		Test	0.926	0.814–1.000	0.833	0.875	0.818	0.947	0.636
T2WI+CE-T1WI	Mean_PCA_ANOVA_SVM	Training	0.964	0.919–0.995	0.944	0.895	0.962	0.895	0.962
		Test	0.909	0.769–1.000	0.800	0.500	0.909	0.833	0.667
T2WI+CE-T1WI+ADC	Z-score_PCA_RFE_AE	Training	0.965	0.920–1.000	0.931	0.842	0.962	0.889	0.944
		Test	0.869	0.727–0.976	0.733	0.625	0.773	0.850	0.500
T2WI+CE-T1WI+SWI	Z-score_PCA_RFE_LDA	Training	0.963	0.905–1.000	0.958	0.947	0.962	0.900	0.981
		Test	0.898	0.761–0.988	0.800	0.750	0.818	0.900	0.600
T2WI+CE-T1WI+CBF	Z-score_PCA_RFE_RF	Training	1.000	1.000–1.000	1.000	1.000	1.000	1.000	1.000
		Test	0.932	0.824–1.000	0.733	0.125	0.955	0.750	0.500
T2WI+CE-T1WI+ADC+SWI	Min–max_PCA_KW_SVM	Training	1.000	1.000–1.000	1.000	1.000	1.000	1.000	1.000
		Test	0.892	0.728–0.994	0.867	0.750	0.909	0.909	0.750
T2WI+CE-T1WI+ADC+CBF	Mean_PCA_Relief_LR	Training	0.555	0.379–0.733	0.736	0.421	0.849	0.500	0.804
		Test	0.881	0.701–1.000	0.900	0.750	0.955	0.913	0.857
T2WI+CE-T1WI+SWI+CBF	Min–max_PCA_RFE_LDA	Training	0.888	0.805–0.957	0.806	0.895	0.774	0.586	0.954
		Test	0.955	0.854–1.000	0.767	1.000	0.682	1.000	0.533
cMRI	Min–max_PCC_Relief_LR	Training	0.833	0.731–0.933	0.778	0.842	0.755	0.552	0.930
		Test	0.921	0.778–1.000	0.733	1.000	0.636	1.000	0.500
aMRI	Mean_PCA_Relief_AB	Training	1.000	1.000–1.000	1.000	1.000	1.000	1.000	1.000
		Test	0.915	0.800–0.993	0.800	0.875	0.773	0.944	0.583
ALL	Z-score_PCA_KW_RF	Training	1.000	1.000–1.000	1.000	1.000	1.000	1.000	1.000
		Test	0.969	0.904–1.000	0.767	0.125	1.000	0.759	1.000

Machine learning technique was expressed as “feature matrix normalization_dimensionality reduction_feature selector_classifier”.

T2WI, T2-weighted imaging; T1WI, T1-weighted imaging; FLAIR, fluid-attenuated inversion recovery; CE-T1WI, contrast-enhanced T1WI; ADC, apparent diffusion coefficient; SWI, susceptibility-weighted imaging; CBV, cerebral blood volume; CBF, cerebral blood flow; cMRI, model developed with all of the conventional MRI; aMRI, model developed with all of the advanced MRI; ALL, model developed with all of the eight sequences; PCC, Pearson’s correlation coefficient; PCA, principal component analysis; RFE, recursive feature elimination; KW, Kruskal–Wallis; LR, logistic regression; LDA, linear discriminant analysis; SVM, support vector machine; AE, auto-encoder, DT, decision tree; RF, random forest; AB, AdaBoost; AUC, area under the curve; ACC, accuracy; SEN, sensibility; SPE, specificity; PPV, positive predictive value; NPV, negative predictive value.

The cMRI showed comparable performance to aMRI when suitable machine learning techniques were employed (DeLong’s test, all *p* > 0.05) (**Table 3**). In models based on a single sequence, the highest AUCs were 0.875–0.915 for cMRI sequences and 0.807–0.926 for aMRI sequences (**Table 2** and **Figure 4**). The model of cMRI yielded a slightly higher AUC than the model of aMRI in the test set (AUC: 0.921 vs. 0.915). When combining limited sequences of cMRI and aMRI, the model of T2WI+CE-T1WI+SWI+CBF reached the highest AUC of 0.955. No statistically significant difference of the highest AUC values between the optimal model (ALL, AUC = 0.969) and other sequence-based models was found (DeLong’s test, all *p* > 0.05) (**Table 3**).

**Table 3:** Results of DeLong’s test of the best models with different classifiers.

Sequence	Highest AUC		Lowest AUC		*p*-Value^a^	*p*-Value^b^	*p*-Value^c^
	Classifier	AUC	Classifier	AUC			
T2WI	AB	0.915	RF	0.847	0.4851	0.4508	0.0527
T1WI	AB	0.875	AB	0.815	0.5602	0.1908	0.0621
FLAIR	AE	0.881	LR	0.767	0.2334	0.1940	**0.0108**
CE-T1WI	LR	0.881	DT	0.744	0.2568	0.1720	**0.0437**
ADC	RF	0.886	SVM	0.727	**0.0118**	0.1837	**0.0144**
SWI	DT	0.869	AB	0.761	0.2292	0.1834	**0.0271**
CBV	AE	0.807	DT	0.722	0.3579	0.1108	**0.0104**
CBF	LR	0.926	DT	0.761	**0.0302**	0.4729	**0.0437**
T2WI+CE-T1WI	SVM	0.909	DT	0.790	0.2944	0.3316	0.0756
T2WI+CE-T1WI+ADC	AE	0.869	DT	0.807	0.4912	0.1319	0.0867
T2WI+CE-T1WI+SWI	LDA	0.898	DT	0.847	0.5049	0.1872	0.5532
T2WI+CE-T1WI+CBF	RF	0.932	SVM	0.847	0.3043	0.5163	0.6795
T2WI+CE-T1WI+ADC+SWI	SVM	0.892	DT	0.790	0.1657	0.2710	1.0000
T2WI+CE-T1WI+ADC+CBF	LR	0.881	DT	0.824	**0.0102**	0.3280	0.0584
T2WI+CE-T1WI+SWI+CBF	LDA	0.955	DT	0.807	0.1121	0.7520	0.8889
cMRI	LR	0.921	DT	0.841	0.2765	0.4145	**0.0077**
aMRI	AB	0.915	DT	0.784	0.1715	0.3243	0.0618
ALL	RF	0.969	DT	0.790	0.0640	–	–

Bold type indicate p < 0.05.

T2WI, T2-weighted imaging; T1WI, T1-weighted imaging; FLAIR, fluid-attenuated inversion recovery; CE-T1WI, contrast-enhanced T1WI; ADC, apparent diffusion coefficient; SWI, susceptibility-weighted imaging; CBV, cerebral blood volume; CBF, cerebral blood flow; cMRI, model developed with all of the conventional MRI; aMRI, model developed with all of the advanced MRI; ALL, model developed with all of the eight sequences; LR, logistic regression; LDA, linear discriminant analysis; SVM, support vector machine; AE, auto-encoder, DT, decision tree; RF, random forest; AB, AdaBoost; AUC, area under the curve.

a p-Value is for the comparison between the best and worst classifiers in the same sequence.

b p-Value is for the comparison of the best classifiers between all sequence-based models (ALL) and other sequence-based models.

c p-Value is for the comparison between the best classifier of all sequence-based models (ALL) and the worst classifier of other sequence-based models.

### Performance of Machine Learning Technique

**Figure 5:**
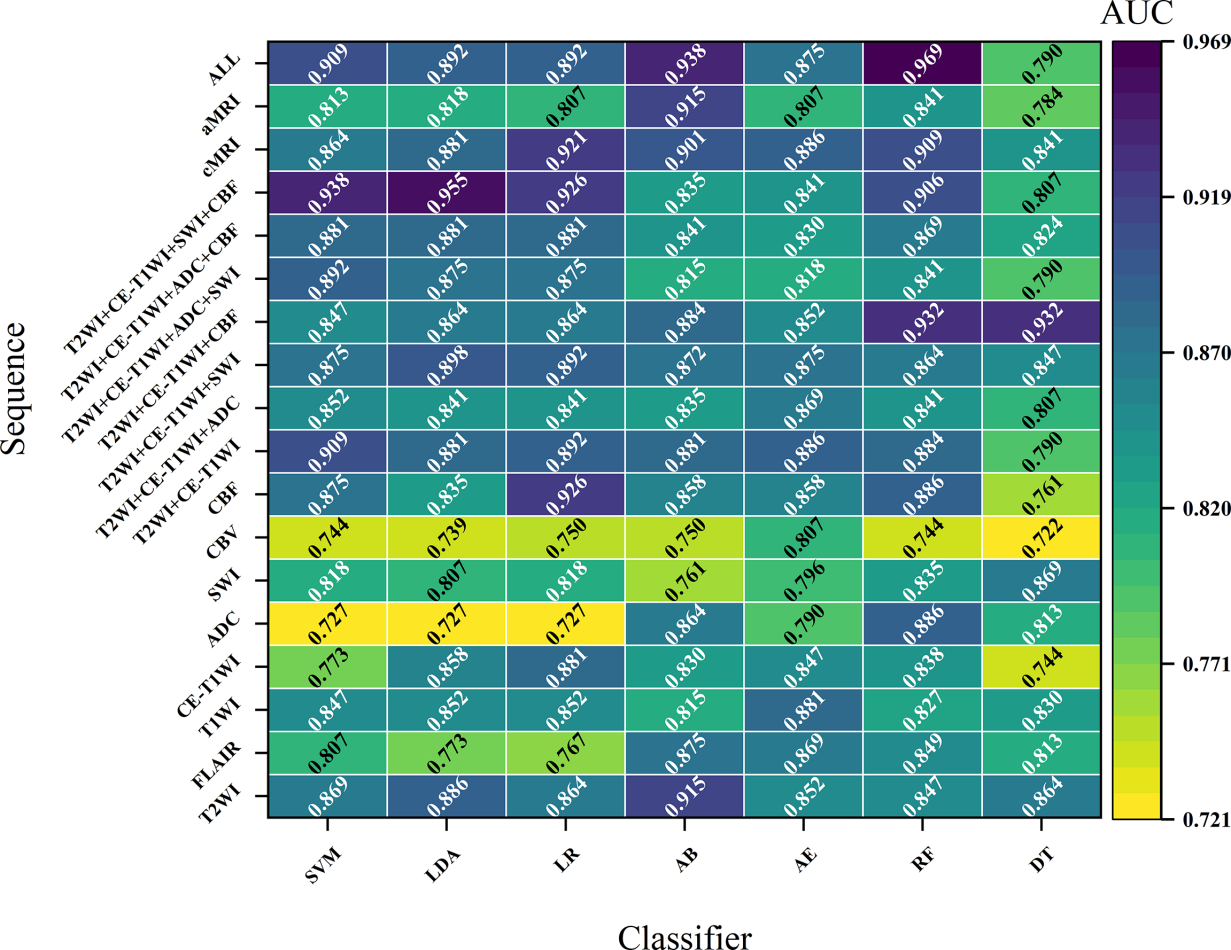
The optimal performance across different sequences and classifiers.

**Figures 2** and **S1** demonstrate the performance of different machine learning techniques. The machine learning pipeline of the optimal model was Z-score_PCA_KW_RF and Z-score_PCA_ANOVA_RF, both with an AUC value of 0.969 (**Figure 2** and **Table S3**). Among the 90 top-five-performing models, the Z-score normalization method outperformed others with darker color lines in **Figure 2** and a higher mean AUC value in **Figure S1**. In the same way, feature sets applying dimensionality reduction with the PCA method had a higher AUC value. **Figure 5** shows the best performance across different sequences and classifiers. The comparison results of the different classifiers are shown in **Table 3**. Of ADC-based models, CBF-based models, and T2WI+CE-T1WI+ADC+CBF-based models, a significant difference could be found in the AUC values between the best classifier and worst classifier (DeLong’s test, *p* < 0.05) (**Table 3**). In contrast to the sequence with a suitable classifier, if the non-optimal classifier was used, the performance of different sequences varied significantly (DeLong’s test, *p* < 0.05) (**Table 3**).

**Figure 6:**
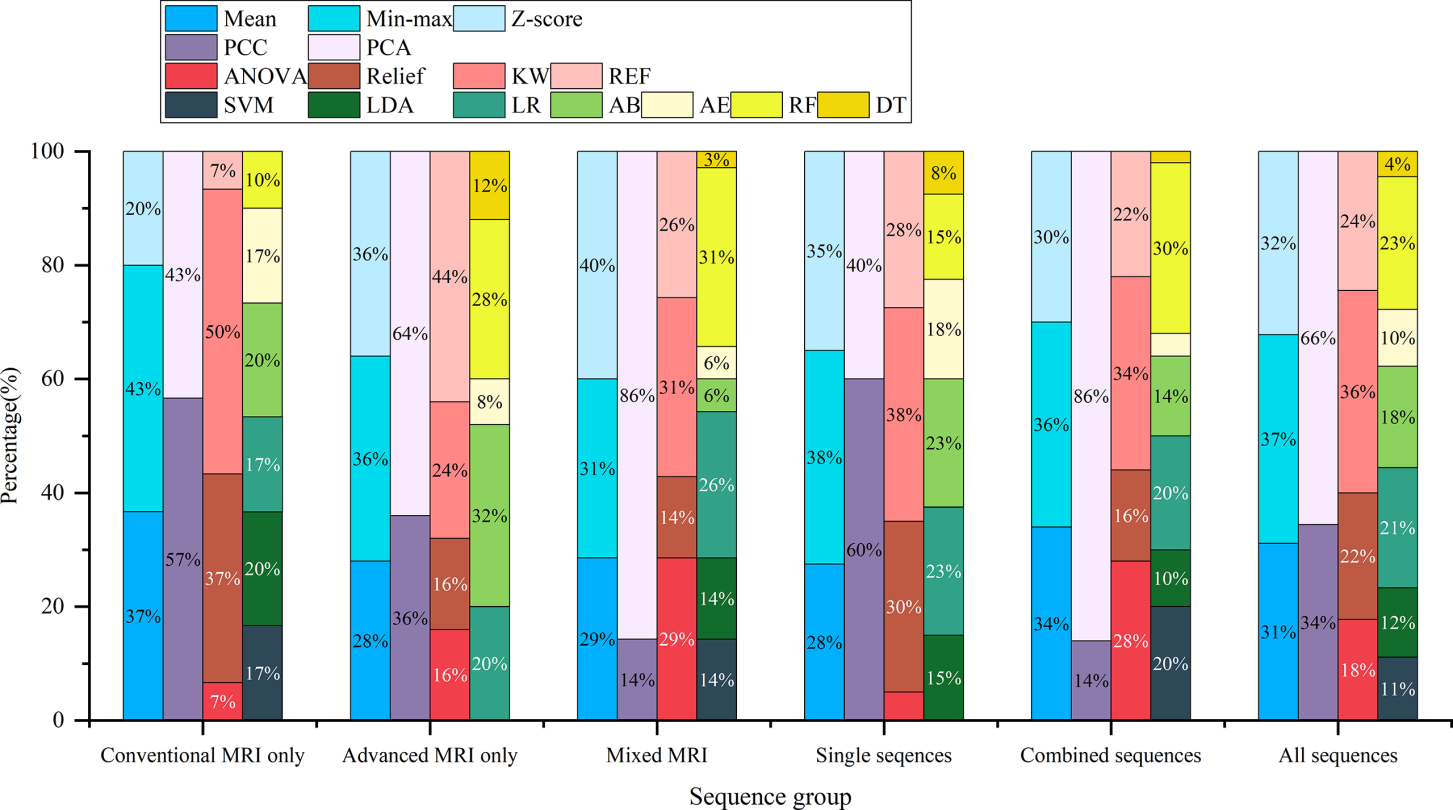
The percentage of machine learning techniques in 90 top-five-performing models of different model categories. “Conventional MRI only” represents models developed only with conventional MRI sequences; “Advanced MRI only” for models only with advanced MRI sequences; “Mixed MRI” for models with both conventional and advanced MRI sequences; “Single sequences” for models with one sequence; “combined sequences” for models with at least two sequences; and “All sequences” for models of all sequence sets.

Among the top-five-performing models, the distribution of machine learning techniques varied considerably in different categories of MRI sequences (**Figures 2**, **6**). PCA was more frequently used in the top-five-performing models (66% of all sequences), especially in the model that simultaneously combined multiple MR images (86%). Feature selector of KW has a higher percentage in both single sequence-based (which have fewer features) and combined sequence-based (which have more features) models.

## Discussion

This study developed and validated various machine learning-based models with radiomics features extracted from multiparametric MRI to predict H3 K27M mutant status in DMG. The model’s performance was compared across different sequences and machine learning techniques. Radiomics models derived from multiparametric MRI performed well in differentiating H3 K27M mutant and wild-type DMGs when a suitable machine learning technique was used (highest AUC: 0.807–0.969). However, the performance of the models can vary significantly regarding different machine learning techniques (DeLong’s test, *p* < 0.05). Generally, the models developed with multi-sequence had a better performance than one with a single sequence. The cMRI-based model showed comparable performance to aMRI (highest AUC: 0.875–0.915 for cMRI, 0.807–0.926 for aMRI).

In line with the previous study, radiomics models based on cMRI could accurately predict the H3 K27M mutant status in DMGs (11–14). As an essential supplement to prior studies, our result also declared that the radiomics model developed with the aMRI, including ADC, SWI, CBV, and CBF, could be qualified for this purpose. Meanwhile, when appropriate machine learning techniques were used, the cMRI and aMRI shared comparable performance (DeLong’s test, *p* > 0.05). A significant difference in ADC, CBV, and CBF values (measured with the freehand regions of interest) has been reported between H3 K27M mutant and wild-type DMGs (28–30). Other studies found that several semantic and semiquantitative features on cMRI could be used to predict H3 K27M mutant status in DMG (31, 32). But other non-radiomics studies using cMRI and DWI to predict H3 K27M mutant status showed converse results (33, 34). Radiomics has been proved to excavate numerous features from medical images, and most of these features are undiscoverable by the naked eye (35, 36). Analyzing medical images with a non-radiomics method may result in a loss of information within images. Wu et al. used radiological features and radiomics features to predict H3 K27M mutant status. Their results showed that the radiomics model performed significantly better than the clinical model (developed with radiological features) (16). The controversial results of non-radiomics studies and the robust results of radiomics studies supported that if the diagnostic information had been sufficiently explored using the radiomics method, the predictive ability of multiparametric MRI could be improved. This has been proved again by our results.

Another important observation was that most models that originated from combined sequences have a better predictive performance, whether the optimal classifier was used (**Figures 2**, **3**) or not (**Figure 5**). Previous studies using a multiparametric MRI-based radiomics model to predict glioma molecular subtype also showed similar results to ours (18, 37). However, only three multiparametric MRI-based radiomics models were established previously and achieved the highest AUC value of 0.920 in the test cohort for H3 K27M mutant status prediction (12, 14, 16). They only make a direct combination of all sequences used, and the performance between single and combined sequences was not compared. Liu et al. developed a machine learning model based on T1WI images only to predict H3 K27M mutant status in DMGs, which yielded the highest AUC value of 0.953 (11). However, the sample size was relatively small (n = 55), and the final model features were slightly overmuch (n = 30). Another radiomics model based on FLAIR images showed an AUC value of 0.903 (13). It is unfair to compare the model’s performance when different datasets were used. Our study compared the model performance based on the same dataset. The results showed that the model had the best predictive power when combined with all sequences (AUC = 0.969). The reason may be that complementary information among multiparametric MRI could provide a more comprehensive understanding of tumor heterogeneity and discriminate more precisely tumor classes. Also noteworthy is that the model combined with limited sequences was sufficient to differentiate H3 K27M-mutant and K27M-wt DMGs, such as the model based on feature sets from T2WI+CE-T1WI+SWI+CBF (AUC = 0.955) and T2WI+CE-T1WI+CBF (AUC = 0.932). This is relevant, as it could guide model application in various clinical circumstances and make it more time-efficient.

According to previous results, the feature selector and classifier were two major determinant factors of radiomics model performance (20–23, 38, 39). When a suitable classifier was used, there was no significant difference in the AUC value of different sequences. Constantly, when an inappropriate classifier was used, both intra-sequence and inter-sequence comparisons yielded a significant difference in AUC values (**Table 3**). For the single sequence-based model, SVM, LDA, and LR classifiers were more frequently to have a lower AUC. The reason may be that the LR and LAD were both linear classifiers, and the SVM used linear kernel function in our study; thus, these classifiers were not flexible enough to fit a non-linear relationship between features and tumor groups. Furthermore, features extracted from a single sequence could only offer limited messages on tumor biological heterogeneity. Of note, the multiparametric MRI-based model with SVM, LDA, and LR demonstrated more favorable results. The prior study used various classifiers (e.g., SVM, RF, and XGBoost) and generated an AUC value of 0.549–0.953, which were lower than ours (AUC = 0.969) (11–15). Several reasons may account for this variety, including patient data, MRI data, and machine learning techniques. Hence, a head-to-head comparison may be more reliable to reveal the influence of these factors and determine optimal models when different image data are available.

Apart from the feature selector and classifier, our results revealed that the feature matrix normalization and dimensionality-reduction method also played a non-negligible role in model performance evaluation (**Figure S1**). The previous study focused on H3 K27M mutant status prediction, which rarely considered these elements. Two of them made an effort to compare the predictive power of different classifiers and another for different feature selectors (11, 15). The limitation of these studies on model development warrants extra caution in terms of result explanation. Our results demonstrated that the appropriate machine learning techniques mentioned above could vary greatly when various image data were used (**Figure 6**). This reemphasized that both the type of image data used and the employment of machine learning techniques will carry a diverse result. Thus, it is essential and encouraged to seek the optimal machine learning techniques when different image data are used. The compatible combination of medical images with machine learning techniques could maximize and robust the radiomics model’s performance.

There are several limitations in the current study. First, this is a single-center retrospective study, which results in an unavoidable selecting bias and relatively small sample size. The imbalanced proportion of H3 K27M mutant DMG might influence the development of our models. A prospective and multi-institution study is needed for confirming our results. Second, the dataset was randomly split into the training and test cohorts. To reduce the selection bias with this kind of splitting, nest cross-validation may be needed in the future. The third is the lack of extra validation to facilitate the generalization of our findings. Unlike other gliomas, the morbidity of DMG was lower. Furthermore, we analyzed eight MR image sets, which makes it more challenging to match an external validation cohort. Fourth, we did not compare our model with the human reader as recommended by a previous study (40). However, the performance of MRI features evaluated by radiologists with the non-radiomics method was controversial, and the discriminative ability was not as well as ours (highest AUC = 0.872) (28). A prior study showed that the radiomics model was significantly superior to the clinical model (based on radiological features) (16). In this regard, our radiomics model might be superior to human readers, although a head-to-head comparison needs to be implemented in the future. Finally, the performance of deep learning algorithms was not evaluated and compared in our study. Deep learning algorithms have been widely used in glioma molecular subtype prediction (41–45). However, deep learning usually needs a huge amount of dataset, such as hundreds or thousands of cases, and the dataset is limited for our approach. More datasets would be collected, and deep-learning algorithms would be compared to classical machine learning algorithms in the future.

## Conclusion

Our results indicated that the H3 K27M mutant status of DMG can be effectively predicted with multiparametric MRI radiomics models. However, the performance of models varies significantly across different machine learning techniques and sequences used.

## Data Availability Statement

The original contributions presented in the study are included in the article/**Supplementary Material**, further inquiries can be directed to the corresponding author.

## Ethics Statement

The studies involving human participants were reviewed and approved by the Ethical Committee of the First Affiliated Hospital of Fujian Medical University. Written informed consent from the participants’ legal guardian/next of kin was not required to participate in this study in accordance with the national legislation and the institutional requirements.

## Author Contributions

WG: writing, study design, and data analysis. DS: study design and data analysis. ZX: data analysis. XL and FW: data collection. YS: model development and statistical analysis. DC: study design, data analysis, and revisions to the manuscript. All authors contributed to the article and approved the submitted version.

## Funding

This study has received funding from the Leading Project of the Department of Science and Technology of Fujian Province (No. 2020Y0025), the National Natural Science Foundation of China (No. 82071869), the Joint Funds of the Innovation of Science and Technology of Fujian Province (No. 2019Y9115), and the Young and Middle-aged Key Personnel Training Project of Fujian Provincial Health Commission (No.2021GGA025).

## Conflict of Interest

Author YS was employed by Siemens Healthineers Ltd.

The remaining authors declare that the research was conducted in the absence of any commercial or financial relationships that could be construed as a potential conflict of interest.

## Publisher’s Note

All claims expressed in this article are solely those of the authors and do not necessarily represent those of their affiliated organizations, or those of the publisher, the editors and the reviewers. Any product that may be evaluated in this article, or claim that may be made by its manufacturer, is not guaranteed or endorsed by the publisher.

## Abbreviations

AB, AdaBoost; ADC, apparent diffusion coefficient; AE, auto-encoder; AUC, area under the curve; CBF, cerebral blood flow; CBV, cerebral blood volume; CE-T1WI, contrast-enhanced T1-weighted imaging; DMG, diffuse midline glioma; DSC-PWI, dynamic susceptibility contrast perfusion-weighted imaging; DT, decision tree; DWI, diffusion-weighted imaging; FLAIR, fluid-attenuated inversion recovery; ICC, intraclass correlation coefficient; KW, Kruskal–Wallis; LDA, linear discriminant analysis; LR, logistic regression; PCA, principal component analysis; PCC, Pearson’s correlation coefficient; RF, random forest; RFE, recursive feature elimination; SVM, support vector machine; SWI, susceptibility-weighted imaging; T1WI, T1-weighted imaging; T2WI, T2-weighted imaging; VOI, volume of interest.
