# Supplementary material for: Multiparametric MRI-Based Radiomics Model for Predicting H3 K27M Mutant Status in Diffuse Midline Glioma: A Comparative Study Across Different Sequences and Machine Learning Techniques
Source: Front Oncol. 2022 Mar 3;12:796583. doi: 10.3389/fonc.2022.796583 (PMC8928064; doi:10.3389/fonc.2022.796583)

Supplementary Material 1

**Table S1.** MRI protocols of the presented study

| Sequence | TR/TE (ms) | FA (º) | FOV (mm) | Matrix | ST (mm) |
| --- | --- | --- | --- | --- | --- |
| T2WI | 6000/96-125 | 90-150 | 220×220 | 320-384×320-384 | 5.0 |
| T1WI | 250/2.5 | 70 | 220×220 | 256-320×205-256 | 5.0 |
| FLAIR | 8500-9000/81-94 | 150 | 220×199 | 256-320×203-232 | 5.0 |
| DWI | 4600-8200/65-102 | 90-180 | 220×220 | 192×192 | 5.0 |
| SWI | 27/20 | 15 | 230-240×172-187 | 256×182-192 | 1.5-2.0 |
| DSC-PWI | 1500-1600/30 | 90 | 230×230 | 128×128 | 5.0 |

Note: T2WI, T2-weighted imaging; T1WI, T1-weighted imaging; FLAIR, fluid-attenuated inversion recovery; DWI, diffusion-weighted imaging; SWI, susceptibility-weighted imaging; DSC-PWI, dynamic susceptibility contrast perfusion-weighted imaging; TR: repetition time; TE, echo time; FA, flip angle; FOV, field of view; ST, slice thickness

**Table S2.** Radiomics features extracted in this study

| **Feature category** | **Feature name** |
| --- | --- |
| **First-order statistics features**  **(n = 18)** | 10 Percentile, 90 Percentile, Energy, Entropy, Interquartile Range, Kurtosis, Maximum, Mean, Mean Absolute Deviation, Median, Minimum, Range, Robust Mean Absolute Deviation, Root Mean Squared |
| **Shape-based features**  **(n = 14)** | Elongation, Flatness, Least Axis Length, Major Axis Length, Maximum 2D Diameter Column, Maximum 2D Diameter Row, Maximum 2D Diameter Slice, Maximum 3D Diameter, Mesh Volume, Minor Axis Length, Sphericity, Surface Area, Surface Volume Ratio, Voxel Volume |
| **Gray-level co-occurrence matrices (GLCM) features**  **(n = 24)** | Autocorrelation, Cluster Prominence, Cluster Shade, Cluster Tendency, Contrast, Correlation, Difference Average, Difference Entropy, Difference Variance, Id, Idm, Idmn, Idn, Imc1, Imc2, Inverse Variance, Joint Average, Joint Energy, Joint Entropy, MCC, Maximum Probability, Sum Average, Sum Entropy, Sum Squares |
| **Gray-level run-length matrices (GLRLM) features**  **(n = 16)** | Gray Level Non-Uniformity, Gray Level Non-Uniformity Normalized, Gray Level Variance, High Gray Level Run Emphasis, Long Run Emphasis, Long Run High Gray Level Emphasis, Long Run Low Gray Level Emphasis, Low Gray Level Run Emphasis, Run Entropy, Run Length Non-Uniformity, Run Length Non-Uniformity Normalized, Run Percentage, Run Variance, Short Run Emphasis, Short Run High Gray Level Emphasis, Short Run Low Gray Level Emphasis |
| **Gray-level size zone matrices (GLSZM) features**  **(n = 16)** | Gray Level Non-Uniformity, Gray Level Non-Uniformity Normalized, Gray Level Variance, High Gray Level Zone Emphasis, Large Area Emphasis, Large Area High Gray Level Emphasis, Large Area Low Gray Level Emphasis, Low Gray Level Zone Emphasis, Size Zone Non-Uniformity, Size Zone Non-Uniformity Normalized, Small Area Emphasis, Small Area High Gray Level Emphasis, Small Area Low Gray Level Emphasis, Zone Entropy, Zone Percentage, Zone Variance |
| **Gray-level dependence matrices (GLDM) features**  **(n = 14)** | Dependence Entropy, Dependence Non-Uniformity, Dependence Non-Uniformity Normalized, Dependence Variance, Gray Level Non-Uniformity, Gray Level Variance, High Gray Level Emphasis, Large Dependence Emphasis, Large Dependence High Gray Level Emphasis, Large Dependence Low Gray Level Emphasis, Low Gray Level Emphasis, Small Dependence Emphasis, Small Dependence High Gray Level Emphasis, Small Dependence Low Gray Level Emphasis |
| **Table S2** (continued) | |
| **Feature category** | **Feature name** |
| **Neighborhood gray-tone difference matrices (NGTDM) features**  **(n = 5)** | Busyness, Coarseness, Complexity, Contrast, Strength |

Note: 2D = two dimension; Id = Inverse Difference; Idm = Inverse Difference Moment; Idmn = Inverse Difference Moment Normalized; Idn = Inverse Difference Normalized; Imc = Informal Measure of Correlation; MCC = Maximal Correlation Coefficient

**Table S3.** The top-five-model of different sequences or sequences combinations

| Sequence | Machine learning technique | Training | |  | Test | |
| --- | --- | --- | --- | --- | --- | --- |
|  |  | AUC | 95%CI |  | AUC | 95%CI |
| T2WI | Min-max_PCA_RFE_AB | 1.000 | 1.000-1.000 |  | 0.915 | 0.769-1.000 |
|  | Mean_PCA_KW_AB | 1.000 | 1.000-1.000 |  | 0.903 | 0.761-1.000 |
|  | Min-max_PCA_KW_AB | 1.000 | 1.000-1.000 |  | 0.903 | 0.761-1.000 |
|  | Mean_PCA_KW_LDA | 0.842 | 0.735-0.933 |  | 0.886 | 0.735-0.995 |
|  | Min-max_PCA_KW_LDA | 0.842 | 0.735-0.933 |  | 0.886 | 0.735-0.995 |
| **Table S3** (continued) | | | | | | |
| Sequence | Machine learning technique | Training | |  | Test | |
|  |  | AUC | 95%CI |  | AUC | 95%CI |
| T1WI | Z-score_PCC_KW_AE | 0.767 | 0.631-0.890 |  | 0.881 | 0.733-0.984 |
|  | Min-max_PCC_KW_LR | 0.793 | 0.661-0.904 |  | 0.852 | 0.690-0.976 |
|  | Mean_PCC_KW_LDA | 0.793 | 0.661-0.902 |  | 0.852 | 0.690-0.976 |
|  | Min-max_PCC_KW_LDA | 0.793 | 0.661-0.902 |  | 0.852 | 0.690-0.976 |
|  | Z-score_PCC_KW_LDA | 0.793 | 0.661-0.902 |  | 0.852 | 0.690-0.976 |
| FLAIR | Mean_PCC_Relief_AB | 1.000 | 1.000-1.000 |  | 0.875 | 0.722-0.984 |
|  | Z-score_PCC_Relief_AB | 1.000 | 1.000-1.000 |  | 0.875 | 0.722-0.984 |
|  | Min-max_PCC_Relief_AE | 0.657 | 0.519-0.801 |  | 0.869 | 0.732-0.977 |
|  | Mean_PCC_Relief_AE | 0.629 | 0.469-0.779 |  | 0.864 | 0.675-1.000 |
|  | Z-score_PCC_Relief_AE | 0.651 | 0.504-0.790 |  | 0.852 | 0.664-0.989 |
| CE-T1WI | Min-max_PCC_Relief_LR | 0.780 | 0.669-0.883 |  | 0.881 | 0.733-0.984 |
|  | Min-max_PCC_Relief_LDA | 0.622 | 0.485-0.740 |  | 0.858 | 0.693-0.975 |
| **Table S3** (continued) | | | | | | |
| Sequence | Machine learning technique | Training | |  | Test | |
|  |  | AUC | 95%CI |  | AUC | 95%CI |
|  | Mean_PCC_Relief_LR | 0.681 | 0.557-0.800 |  | 0.852 | 0.680-0.976 |
|  | Min-max_PCC_Relief_AE | 0.802 | 0.699-0.901 |  | 0.847 | 0.689-0.968 |
|  | Z-score_PCC_Relief_LR | 0.703 | 0.577-0.821 |  | 0.847 | 0.675-0.973 |
| ADC | Min-max_PCC_RFE_RF | 1.000 | 1.000-1.000 |  | 0.886 | 0.718-1.000 |
|  | Min-max_PCC_RFE_AB | 1.000 | 1.000-1.000 |  | 0.864 | 0.701-0.975 |
|  | Min-max_PCC_RFE_DT | 1.000 | 1.000-1.000 |  | 0.813 | 0.625-1.000 |
|  | Mean_PCA_KW_AB | 1.000 | 1.000-1.000 |  | 0.790 | 0.611-0.938 |
|  | Min-max_PCA_KW_AB | 1.000 | 1.000-1.000 |  | 0.790 | 0.611-0.938 |
| SWI | Mean_PCC_RFE_DT | 1.000 | 1.000-1.000 |  | 0.869 | 0.694-0.979 |
|  | Z-score_PCC_RFE_DT | 1.000 | 1.000-1.000 |  | 0.869 | 0.694-0.979 |
|  | Mean_PCC_RFE_RF | 1.000 | 1.000-1.000 |  | 0.835 | 0.671-0.965 |
|  | Z-score_PCC_RFE_RF | 1.000 | 1.000-1.000 |  | 0.835 | 0.671-0.965 |
| **Table S3** (continued) | | | | | | |
| Sequence | Machine learning technique | Training | |  | Test | |
|  |  | AUC | 95%CI |  | AUC | 95%CI |
|  | Z-score_PCA_KW_RF | 1.000 | 1.000-1.000 |  | 0.818 | 0.629-0.950 |
| CBV | Mean_PCA_Relief_AE | 0.640 | 0.490-0.779 |  | 0.807 | 0.585-0.980 |
|  | Z-score_PCA_RFE_AE | 0.752 | 0.622-0.878 |  | 0.778 | 0.556-0.957 |
|  | Min-max_PCA_Relief_AB | 1.000 | 1.000-1.000 |  | 0.750 | 0.548-0.914 |
|  | Mean_PCC_KW_LR | 0.868 | 0.779-0.950 |  | 0.750 | 0.520-0.944 |
|  | Min-max_PCC_KW_LR | 0.852 | 0.753-0.939 |  | 0.750 | 0.519-0.944 |
| CBF | Z-score_PCA_RFE_LR | 0.924 | 0.844-0.983 |  | 0.926 | 0.814-1.000 |
|  | Z-score_PCA_ANOVA_LR | 0.957 | 0.904-0.993 |  | 0.921 | 0.776-1.000 |
|  | Z-score_PCA_KW_LR | 0.984 | 0.953-1.000 |  | 0.909 | 0.729-1.000 |
|  | Z-score_PCA_RFE_RF | 1.000 | 1.000-1.000 |  | 0.886 | 0.740-0.989 |
|  | Z-score_PCA_ANOVA_RF | 1.000 | 1.000-1.000 |  | 0.886 | 0.727-1.000 |
| T2WI+CE-T1WI | Mean_PCA_ANOVA_SVM | 0.964 | 0.919-0.995 |  | 0.909 | 0.769-1.000 |
| **Table S3** (continued) | | | | | | |
| Sequence | Machine learning technique | Training | |  | Test | |
|  |  | AUC | 95%CI |  | AUC | 95%CI |
|  | Min-max_PCA_ANOVA_SVM | 0.964 | 0.919-0.995 |  | 0.909 | 0.769-1.000 |
|  | Mean_PCA_RFE_SVM | 0.964 | 0.914-0.999 |  | 0.898 | 0.752-1.000 |
|  | Mean_PCA_KW_SVM | 0.947 | 0.887-0.993 |  | 0.898 | 0.761-0.991 |
|  | Min-max_PCA_KW_SVM | 0.947 | 0.887-0.993 |  | 0.898 | 0.761-0.991 |
| T2WI+CE-T1WI+ADC | Z-score_PCA_RFE_AE | 0.965 | 0.920-1.000 |  | 0.869 | 0.727-0.976 |
|  | Z-score_PCA_RFE_SVM | 0.941 | 0.879-0.989 |  | 0.852 | 0.652-1.000 |
|  | Mean_PCC_ANOVA_RF | 1.000 | 1.000-1.000 |  | 0.841 | 0.652-0.976 |
|  | Min-max_PCC_ANOVA_RF | 1.000 | 1.000-1.000 |  | 0.841 | 0.652-0.976 |
|  | Z-score_PCC_ANOVA_RF | 1.000 | 1.000-1.000 |  | 0.841 | 0.652-0.976 |
| T2WI+CE-T1WI+SWI | Z-score_PCA_RFE_LDA | 0.963 | 0.905-1.000 |  | 0.898 | 0.761-0.988 |
|  | Z-score_PCA_RFE_LR | 0.958 | 0.892-1.000 |  | 0.892 | 0.750-0.986 |
|  | Z-score_PCA_ANOVA_SVM | 0.974 | 0.928-1.000 |  | 0.875 | 0.714-0.979 |
| **Table S3** (continued) | | | | | | |
| Sequence | Machine learning technique | Training | |  | Test | |
|  |  | AUC | 95%CI |  | AUC | 95%CI |
|  | Min-max_PCC_Relief_AE | 0.652 | 0.515-0.769 |  | 0.875 | 0.722-0.986 |
|  | Mean_PCC_ANOVA_AB | 1.000 | 1.000-1.000 |  | 0.872 | 0.720-0.985 |
| T2WI+CE-T1WI+CBF | Z-score_PCA_RFE_RF | 1.000 | 1.000-1.000 |  | 0.932 | 0.824-1.000 |
|  | Z-score_PCA_RFE_DT | 1.000 | 1.000-1.000 |  | 0.932 | 0.853-1.000 |
|  | Z-score_PCA_KW_RF | 1.000 | 1.000-1.000 |  | 0.926 | 0.804-1.000 |
|  | Min-max_PCA_ANOVA_RF | 1.000 | 1.000-1.000 |  | 0.909 | 0.760-0.992 |
|  | Mean_PCA_ANOVA_RF | 1.000 | 1.000-1.000 |  | 0.909 | 0.760-0.992 |
| T2WI+CE-T1WI+ADC+SWI | Min-max_PCA_KW_SVM | 1.000 | 1.000-1.000 |  | 0.892 | 0.728-0.994 |
|  | Min-max_PCA_KW_LR | 0.999 | 0.994-1.000 |  | 0.875 | 0.712-1.000 |
|  | Min-max_PCA_KW_LDA | 0.937 | 0.880-0.981 |  | 0.875 | 0.705-0.993 |
|  | Mean_PCA_KW_LDA | 0.937 | 0.880-0.981 |  | 0.875 | 0.705-0.993 |
|  | Mean_PCA_KW_LR | 0.989 | 0.969-1.000 |  | 0.864 | 0.683-0.993 |
| **Table S3** (continued) | | | | | | |
| Sequence | Machine learning technique | Training | |  | Test | |
|  |  | AUC | 95%CI |  | AUC | 95%CI |
| T2WI+CE-T1WI+ADC+CBF | Mean_PCA_Relief_LR | 0.555 | 0.379-0.733 |  | 0.881 | 0.701-1.000 |
|  | Mean_PCA_Relief_LDA | 0.555 | 0.379-0.733 |  | 0.881 | 0.701-1.000 |
|  | Mean_PCA_Relief_SVM | 0.555 | 0.379-0.733 |  | 0.881 | 0.701-1.000 |
|  | Mean_PCA_KW_LR | 0.928 | 0.863-0.980 |  | 0.875 | 0.643-1.000 |
|  | Min-max_PCA_KW_LR | 0.928 | 0.863-0.980 |  | 0.875 | 0.643-1.000 |
| T2WI+CE-T1WI+SWI+CBF | Min-max_PCA_RFE_LDA | 0.888 | 0.805-0.957 |  | 0.955 | 0.854-1.000 |
|  | Min-max_PCA_RFE_SVM | 0.880 | 0.786-0.957 |  | 0.938 | 0.826-1.000 |
|  | Z-score_PCA_ANOVA_LR | 0.981 | 0.948-1.000 |  | 0.926 | 0.806-1.000 |
|  | Mean_PCA_KW_LR | 0.969 | 0.928-0.998 |  | 0.926 | 0.799-1.000 |
|  | Min-max_PCA_KW_LR | 0.969 | 0.928-0.998 |  | 0.926 | 0.799-1.000 |
| cMRI | Min-max_PCC_Relief_LR | 0.833 | 0.731-0.933 |  | 0.921 | 0.778-1.000 |
|  | Min-max_PCA_KW_RF | 1.000 | 1.000-1.000 |  | 0.909 | 0.787-1.000 |
| **Table S3** (continued) | | | | | | |
| Sequence | Machine learning technique | Training | |  | Test | |
|  |  | AUC | 95%CI |  | AUC | 95%CI |
|  | Mean_PCA_KW_RF | 1.000 | 1.000-1.000 |  | 0.906 | 0.765-0.990 |
|  | Z-score_PCA_KW_RF | 1.000 | 1.000-1.000 |  | 0.903 | 0.756-1.000 |
|  | Mean_PCC_KW_AB | 1.000 | 1.000-1.000 |  | 0.901 | 0.761-1.000 |
| aMRI | Mean_PCA_Relief_AB | 1.000 | 1.000-1.000 |  | 0.915 | 0.800-0.993 |
|  | Min-max_PCA_RFE_AB | 1.000 | 1.000-1.000 |  | 0.898 | 0.696-1.000 |
|  | Min-max_PCA_Relief_RF | 1.000 | 1.000-1.000 |  | 0.841 | 0.667-0.977 |
|  | Mean_PCA_ANOVA_AB | 1.000 | 1.000-1.000 |  | 0.824 | 0.603-0.981 |
|  | Min-max_PCA_ANOVA_AB | 1.000 | 1.000-1.000 |  | 0.824 | 0.603-0.981 |
| ALL | Z-score_PCA_KW_RF | 1.000 | 1.000-1.000 |  | 0.969 | 0.904-1.000 |
|  | Z-score_PCA_ANOVA_RF | 1.000 | 1.000-1.000 |  | 0.969 | 0.904-1.000 |
|  | Min-max_PCA_Relief_RF | 1.000 | 1.000-1.000 |  | 0.940 | 0.824-1.000 |
|  | Z-score_PCA_ANOVA_AB | 1.000 | 1.000-1.000 |  | 0.938 | 0.817-1.000 |
| **Table S3** (continued) | | | | | | |
| Sequence | Machine learning technique | Training | |  | Test | |
|  |  | AUC | 95%CI |  | AUC | 95%CI |
|  | Z-score_PCA_RFE_RF | 1.000 | 1.000-1.000 |  | 0.926 | 0.807-1.000 |

Note: T2WI, T2-weighted imaging; T1WI, T1-weighted imaging; FLAIR, fluid-attenuated inversion recovery; CE-T1WI, contrast-enhanced T1WI; ADC, apparent diffusion coefficient; SWI, susceptibility-weighted imaging; CBV, cerebral blood volume; CBF, cerebral blood flow; PCC, Pearson correlation coefficient; PCA, principal component analysis; ANOVA, analysis of variance; RFE, recursive feature elimination; KW, Kruskal Wallis; LR, logistic regression; LDA, linear discriminant analysis; SVM, support vector machine; AE, auto-encoder, DT, decision tree; RF, random forest; AB, AdaBoost; AUC, area under the curve; CI, confidence interval

**Figure S1.** Box and whisker plots illustrated the AUC values of top-five-performing models of different machine learning techniques. (A) Feature matrix normalization. (B) Dimensionality reduction. (C) Feature selector. (D) Classifier.


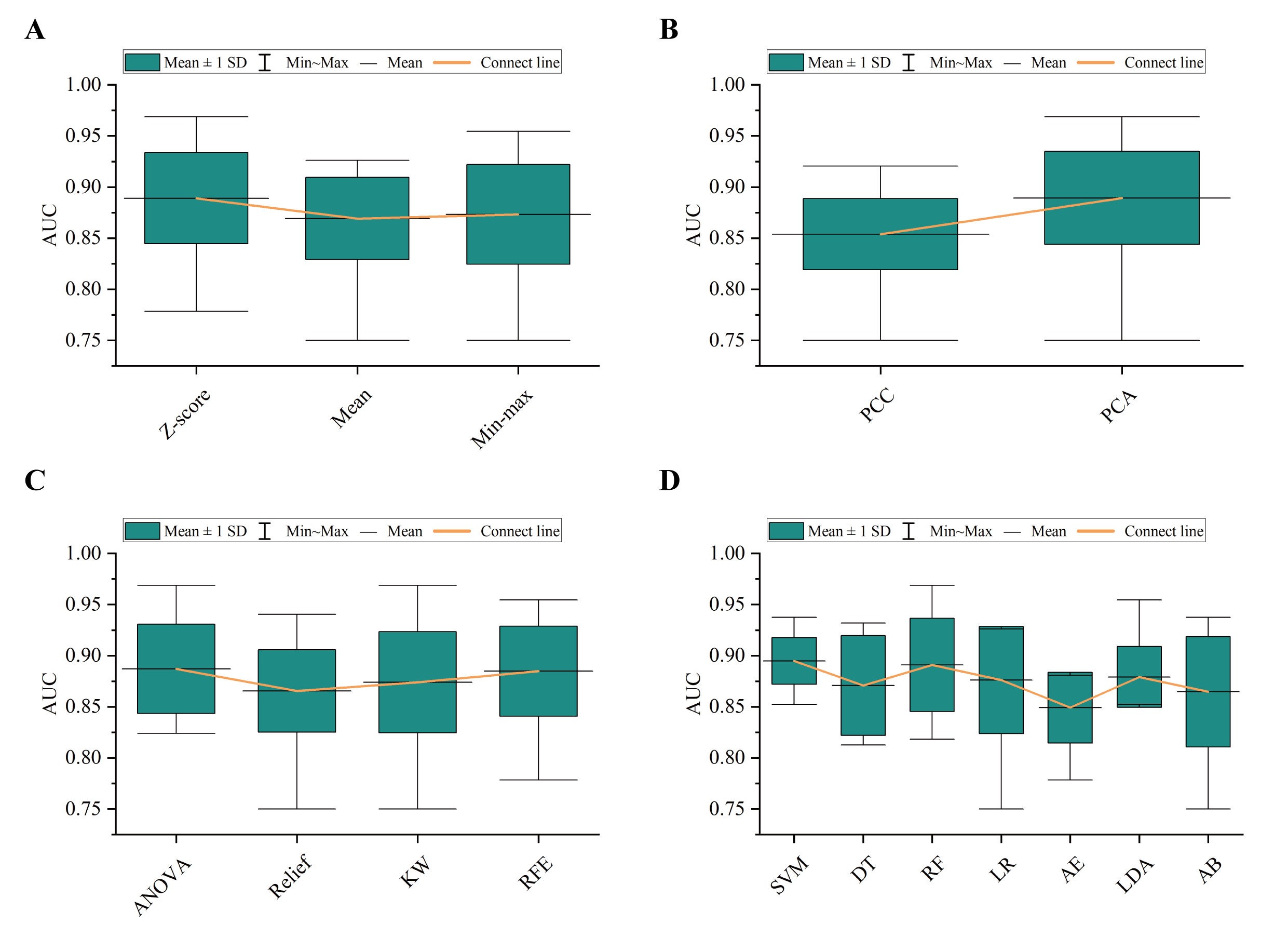

Supplement: Supplementary file 1 [file DataSheet_1.docx]
