# Supplementary material for: Multiparametric MRI-Based Radiomics Model for Predicting H3 K27M Mutant Status in Diffuse Midline Glioma: A Comparative Study Across Different Sequences and Machine Learning Techniques
Source: Front Oncol. 2022 Mar 3;12:796583. doi: 10.3389/fonc.2022.796583 (PMC8928064; doi:10.3389/fonc.2022.796583)

Supplementary Material 2

Table S1. The top-five-model based on original radiomics features of different sequences or sequences combinations

| Sequence | Machine learning technique | Training | |  | Test | |
| --- | --- | --- | --- | --- | --- | --- |
|  |  | AUC | 95%CI |  | AUC | 95%CI |
| T2WI | Min-max_PCA_Relief_SVM | 0.483 | 0.327-0.649 |  | 0.881 | 0.679-1.000 |
|  | Mean_PCA_RFE_AE | 0.470 | 0.326-0.613 |  | 0.864 | 0.701-0.984 |
|  | Mean_PCC_Relief_AB | 1.000 | 1.000-1.000 |  | 0.830 | 0.636-0.966 |
|  | Z-score_PCC_Relief_AB | 1.000 | 1.000-1.000 |  | 0.830 | 0.636-0.966 |
|  | Z-score_PCC_KW_AE | 0.663 | 0.520-0.799 |  | 0.796 | 0.609-0.944 |
| T1WI | Min-max_PCC_RFE_DT | 1.000 | 1.000-1.000 |  | 0.869 | 0.716-0.979 |
|  | Mean_PCC_ANOVA_DT | 1.000 | 1.000-1.000 |  | 0.869 | 0.716-0.979 |
|  | Min-max_PCC_ANOVA_DT | 1.000 | 1.000-1.000 |  | 0.869 | 0.716-0.979 |
|  | Z-score_PCC_ANOVA_DT | 1.000 | 1.000-1.000 |  | 0.869 | 0.716-0.979 |
|  | Mean_PCC_RFE_AE | 0.598 | 0.431-0.773 |  | 0.869 | 0.693-1.000 |
| **Table S1** (continued) | | | | | | |
| Sequence | Machine learning technique | Training | |  | Test | |
|  |  | AUC | 95%CI |  | AUC | 95%CI |
| FLAIR | Mean_PCC_KW_AE | 0.671 | 0.533-0.819 |  | 0.909 | 0.783-1.000 |
|  | Mean_PCC_Relief_DT | 1.000 | 1.000-1.000 |  | 0.864 | 0.762-0.952 |
|  | Z-score_PCC_Relief_DT | 1.000 | 1.000-1.000 |  | 0.864 | 0.762-0.952 |
|  | Min-max_PCC_Relief_RF | 0.980 | 0.933-1.000 |  | 0.858 | 0.681-0.986 |
|  | Z-score_PCC_KW_AE | 0.763 | 0.654-0.874 |  | 0.858 | 0.705-0.992 |
| CE-T1WI | Z-score_PCC_Relief_SVM | 0.420 | 0.277-0.571 |  | 0.915 | 0.796-1.000 |
|  | Mean_PCA_KW_AB | 1.000 | 1.000-1.000 |  | 0.881 | 0.730-0.984 |
|  | Min-max_PCA_KW_AB | 1.000 | 1.000-1.000 |  | 0.881 | 0.730-0.984 |
|  | Z-score_PCA_KW_LDA | 0.957 | 0.879-1.000 |  | 0.875 | 0.751-0.976 |
|  | Z-score_PCA_ANOVA_LDA | 0.957 | 0.887-1.000 |  | 0.875 | 0.751-0.976 |
| ADC | Z-score_PCA_Relief_SVM | 0.493 | 0.323-0.666 |  | 0.796 | 0.639-0.940 |
|  | Min-max_PCC_ANOVA_AE | 0.564 | 0.422-0.715 |  | 0.778 | 0.556-0.963 |
| **Table S1** (continued) | | | | | | |
| Sequence | Machine learning technique | Training | |  | Test | |
|  |  | AUC | 95%CI |  | AUC | 95%CI |
|  | Z-score_PCA_RFE_LDA | 0.899 | 0.806-0.973 |  | 0.756 | 0.530-0.933 |
|  | Z-score_PCC_Relief_LDA | 0.739 | 0.612-0.861 |  | 0.756 | 0.553-0.919 |
|  | Mean_PCC_Relief_LDA | 0.739 | 0.612-0.861 |  | 0.756 | 0.553-0.919 |
| SWI | Z-score_PCA_Relief_LR | 0.741 | 0.615-0.870 |  | 0.841 | 0.660-0.981 |
|  | Mean_PCA_RFE__RF | 1.000 | 1.000-1.000 |  | 0.830 | 0.646-0.957 |
|  | Z-score_PCA_Relief_LDA | 0.762 | 0.609-0.894 |  | 0.784 | 0.563-0.964 |
|  | Mean_PCA_RFE_DT | 1.000 | 1.000-1.000 |  | 0.778 | 0.603-0.913 |
|  | Z-score_PCA_ANOVA_AE | 0.624 | 0.487-0.761 |  | 0.778 | 0.544-0.966 |
| CBV | Mean_PCC_ANOVA_AE | 0.707 | 0.564-0.844 |  | 0.875 | 0.634-1.000 |
|  | Min-max_PCC_Relief_AE | 0.587 | 0.457-0.717 |  | 0.841 | 0.672-0.977 |
|  | Z-score_PCC_ANOVA_AE | 0.769 | 0.627-0.894 |  | 0.767 | 0.504-0.981 |
|  | Mean_PCC_RFE_AE | 0.640 | 0.498-0.783 |  | 0.767 | 0.539-0.963 |
| **Table S1** (continued) | | | | | | |
| Sequence | Machine learning technique | Training | |  | Test | |
|  |  | AUC | 95%CI |  | AUC | 95%CI |
|  | Mean_PCA_Relief_RF | 1.000 | 1.000-1.000 |  | 0.761 | 0.544-0.947 |
| CBF | Mean_PCA_RFE_RF | 1.000 | 1.000-1.000 |  | 0.903 | 0.776-0.993 |
|  | Min-max_PCA_RFE_RF | 1.000 | 1.000-1.000 |  | 0.903 | 0.776-0.993 |
|  | Mean_PCA_KW_RF | 1.000 | 1.000-1.000 |  | 0.898 | 0.771-0.988 |
|  | Min-max_PCA_KW_RF | 1.000 | 1.000-1.000 |  | 0.898 | 0.771-0.988 |
|  | Mean_PCA_ANOVA_RF | 1.000 | 1.000-1.000 |  | 0.898 | 0.771-0.988 |
| T2WI+CE-T1WI | Mean_PCC_Relief_AB | 1.000 | 1.000-1.000 |  | 0.903 | 0.776-0.994 |
|  | Z-score_PCC_Relief_AB | 1.000 | 1.000-1.000 |  | 0.903 | 0.776-0.994 |
|  | Mean_PCC_Relief_AE | 0.654 | 0.512-0.782 |  | 0.886 | 0.751-0.990 |
|  | Min-max_PCA_Relief_AE | 0.507 | 0.356-0.674 |  | 0.875 | 0.715-0.986 |
|  | Min-max_PCA_KW_RF | 1.000 | 1.000-1.000 |  | 0.841 | 0.661-0.971 |
| T2WI+CE-T1WI+ADC | Min-max_PCA_RFE_DT | 1.000 | 1.000-1.000 |  | 0.915 | 0.760-1.000 |
| **Table S1** (continued) | | | | | | |
| Sequence | Machine learning technique | Training | |  | Test | |
|  |  | AUC | 95%CI |  | AUC | 95%CI |
|  | Mean_PCA_ANOVA_DT | 1.000 | 1.000-1.000 |  | 0.915 | 0.760-1.000 |
|  | Min-max_PCA_ANOVA_DT | 1.000 | 1.000-1.000 |  | 0.915 | 0.760-1.000 |
|  | Mean_PCA_ANOVA_RF | 1.000 | 1.000-1.000 |  | 0.909 | 0.696-1.000 |
|  | Min-max_PCA_ANOVA_RF | 1.000 | 1.000-1.000 |  | 0.909 | 0.696-1.000 |
| T2WI+CE-T1WI+SWI | Z-score_PCA_Relief_LR | 0.652 | 0.504-0.793 |  | 0.830 | 0.680-0.965 |
|  | Z-score_PCA_Relief_LDA | 0.654 | 0.508-0.797 |  | 0.824 | 0.670-0.960 |
|  | Z-score_PCA_KW_AE | 0.425 | 0.260-0.595 |  | 0.818 | 0.634-0.963 |
|  | Min-max_PCA_RFE_SVM | 0.842 | 0.693-0.968 |  | 0.801 | 0.528-1.000 |
|  | Mean_PCC_KW_RF | 1.000 | 1.000-1.000 |  | 0.796 | 0.603-0.968 |
| T2WI+CE-T1WI+CBF | Z-score_PCA_RFE_SVM | 0.926 | 0.803-1.000 |  | 0.852 | 0.590-1.000 |
|  | Mean_PCC_Relief_AB | 1.000 | 1.000-1.000 |  | 0.847 | 0.674-0.979 |
|  | Z-score_PCC_Relief_AB | 1.000 | 1.000-1.000 |  | 0.847 | 0.674-0.979 |
| **Table S1** (continued) | | | | | | |
| Sequence | Machine learning technique | Training | |  | Test | |
|  |  | AUC | 95%CI |  | AUC | 95%CI |
|  | Z-score_PCA_ANOVA_SVM | 0.955 | 0.890-1.000 |  | 0.847 | 0.575-1.000 |
|  | Min-max_PCC_Relief_AE | 0.485 | 0.320-0.661 |  | 0.847 | 0.660-0.966 |
| T2WI+CE-T1WI+ADC+SWI | Z-score_PCA_RFE_RF | 1.000 | 1.000-1.000 |  | 0.835 | 0.642-0.982 |
|  | Z-score_PCA_ANOVA_AB | 1.000 | 1.000-1.000 |  | 0.801 | 0.609-0.963 |
|  | Min-max_PCC_RFE_AE | 0.402 | 0.244-0.567 |  | 0.801 | 0.576-0.968 |
|  | Mean_PCC_KW_RF | 1.000 | 1.000-1.000 |  | 0.796 | 0.603-0.968 |
|  | Min-max_PCC_KW_RF | 1.000 | 1.000-1.000 |  | 0.796 | 0.603-0.968 |
| T2WI+CE-T1WI+ADC+CBF | Min-max_PCA_RFE_RF | 1.000 | 1.000-1.000 |  | 0.921 | 0.794-1.000 |
|  | Mean_PCA_RFE_RF | 1.000 | 1.000-1.000 |  | 0.921 | 0.794-1.000 |
|  | Min-max_PCA_RFE_LDA | 0.922 | 0.839-0.981 |  | 0.892 | 0.757-0.988 |
|  | Min-max_PCC_Relief_AE | 0.616 | 0.449-0.777 |  | 0.881 | 0.725-0.983 |
|  | Mean_PCA_ANOVA_LDA | 0.882 | 0.782-0.971 |  | 0.875 | 0.718-0.981 |
| **Table S1** (continued) | | | | | | |
| Sequence | Machine learning technique | Training | |  | Test | |
|  |  | AUC | 95%CI |  | AUC | 95%CI |
| T2WI+CE-T1WI+SWI+CBF | Min-max_PCA_RFE_LR | 0.740 | 0.609-0.865 |  | 0.858 | 0.674-0.993 |
|  | Min-max_PCA_RFE_LDA | 0.739 | 0.605-0.869 |  | 0.858 | 0.670-0.995 |
|  | Mean_PCA_KW_RF | 1.000 | 1.000-1.000 |  | 0.841 | 0.684-0.963 |
|  | Min-max_PCA_KW_RF | 1.000 | 1.000-1.000 |  | 0.841 | 0.684-0.963 |
|  | Mean_PCC_KW_LDA | 0.832 | 0.712-0.933 |  | 0.835 | 0.665-0.960 |
| cMRI | Z-score_PCA_KW_RF | 1.000 | 1.000-1.000 |  | 0.903 | 0.764-1.000 |
|  | Z-score_PCA_ANOVA_AE | 0.704 | 0.555-0.838 |  | 0.898 | 0.744-0.990 |
|  | Z-score_PCA_ANOVA_RF | 1.000 | 1.000-1.000 |  | 0.892 | 0.757-0.986 |
|  | Z-score_PCA_RFE_RF | 1.000 | 1.000-1.000 |  | 0.884 | 0.752-0.979 |
|  | Min-max_PCC_ANOVA_AE | 0.737 | 0.606-0.857 |  | 0.881 | 0.732-1.000 |
| aMRI | Min-max_PCC_RFE_SVM | 0.835 | 0.712-0.945 |  | 0.864 | 0.694-0.985 |
|  | Z-score_PCC_RFE_LR | 0.926 | 0.843-0.990 |  | 0.858 | 0.636-1.000 |
| **Table S1** (continued) | | | | | | |
| Sequence | Machine learning technique | Training | |  | Test | |
|  |  | AUC | 95%CI |  | AUC | 95%CI |
|  | Mean_PCC_RFE_LR | 0.925 | 0.843-0.989 |  | 0.858 | 0.636-1.000 |
|  | Z-score_PCC_RFE_SVM | 0.919 | 0.838-0.986 |  | 0.852 | 0.640-1.000 |
|  | Min-max_PCC_RFE_LR | 0.848 | 0.727-0.952 |  | 0.847 | 0.667-0.972 |
| ALL | Mean_PCC_Relief_AE | 0.747 | 0.603-0.876 |  | 0.943 | 0.808-1.000 |
|  | Z-score_PCC_Relief_AE | 0.745 | 0.596-0.878 |  | 0.903 | 0.768-0.989 |
|  | Mean_PCC_Relief_LDA | 0.603 | 0.459-0.744 |  | 0.903 | 0.760-1.000 |
|  | Z-score_PCC_Relief_LDA | 0.603 | 0.459-0.744 |  | 0.903 | 0.760-1.000 |
|  | Mean_PCC_Relief_LR | 0.597 | 0.455-0.742 |  | 0.903 | 0.760-1.000 |

Note: T2WI, T2-weighted imaging; T1WI, T1-weighted imaging; FLAIR, fluid-attenuated inversion recovery; CE-T1WI, contrast-enhanced T1WI; ADC, apparent diffusion coefficient; SWI, susceptibility-weighted imaging; CBV, cerebral blood volume; CBF, cerebral blood flow; PCC, Pearson correlation coefficient; PCA, principal component analysis; ANOVA, analysis of variance; RFE, recursive feature elimination; KW, Kruskal Wallis; LR, logistic regression; LDA, linear discriminant analysis; SVM, support vector machine; AE, auto-encoder, DT, decision tree; RF, random forest; AB, AdaBoost; AUC, area under the curve; CI, confidence interval

Figure S1. The optimal performance across different sequences and classifiers based on original radiomics features





Figure S2. The machine learning pipelines and performance of top-five-performing models of different sequences. (A) The details of machine learning techniques used in top-five-performing models developed with original radiomics features of different sequences and the performance on the test cohort. (B) The performance of top-five-performing models based on original radiomics features on the training, test cohort, and their deviation (AUC_Delta = AUC_Training – AUC_Test). (C) The performance of top-five-performing models based on original and wavelet radiomics features on the training, test cohort, and their deviation (AUC_Delta = AUC_Training – AUC_Test)


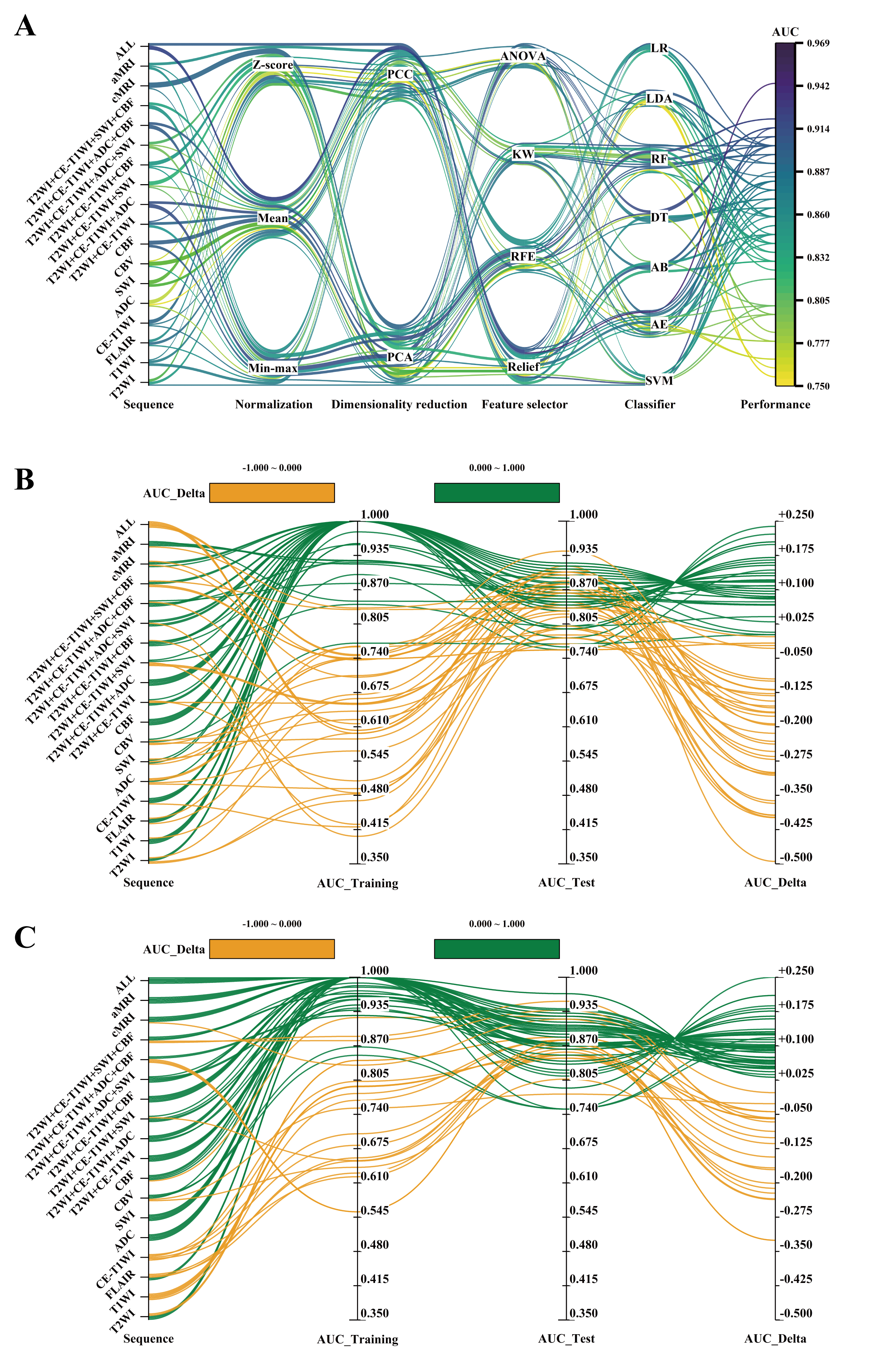

Supplement: Supplementary file 2 [file DataSheet_2.docx]
